# Colpocleisis as an obliterative surgery for pelvic organ prolapse: A single-center experience

**DOI:** 10.1097/MD.0000000000046411

**Published:** 2026-01-23

**Authors:** Çağlanur Yildiz, Ayşe Zehra Özdemir, Burak Barutçu, Fatmanur Mollahüseyinoğlu Küllaç, İsmail Akkafa, Kübra Baki Erin, Recep Erin

**Affiliations:** a Department of Obstetrics and Gynecology, Gölbaşi Government Hospital, Adiyaman, Türkiye; b Department of Obstetrics and Gynecology, Medical School, Ondokuz Mayis University, Samsun, Türkiye; c Department of Obstetrics and Gynecology, Bafra Government Hospital, Samsun, Türkiye; d Trabzon Kanuni Health Practice and Research Center, University of Health Sciences, Trabzon, Türkiye.

**Keywords:** colpocleisis, elderly, obliterative surgery, pelvic organ prolapse, quality of life, regret

## Abstract

**Background:**

Pelvic organ prolapse (POP) is a common condition, and its incidence is anticipated to increase with the aging population. When surgical intervention is required for a POP, the surgeon must select between reconstructive and obliterative techniques. Colpocleisis is a proficient surgical procedure for geriatric patients with POP and various comorbidities, particularly for those who do not desire to maintain vaginal functionality for intercourse. This study seeks to assess the experience of a single center with the colpocleisis procedure.

**Methods:**

This study retrospectively analyzed 58 cases of colpocleisis performed at the Ondokuz Mayis University Faculty of Medicine from July 21, 2015, to January 17, 2025. This encompassed the patients’ preoperative and postoperative medical histories as well as perioperative outcomes. The Patient Global Impression-Improvement questionnaire was utilized to assess perceived efficacy. We assessed self-reported quality of life utilizing the Pelvic Floor Distress Inventory – questionnaire 20. We evaluated regret by posing an additional inquiry: “Do you regret opting for vaginal closure surgery for prolapse (Yes/No)?”

**Results:**

The mean age of the patients was 75.55 ± 8.21. POP stage 3 was diagnosed in 93.1% of patients. The mean postoperative follow-up period was 2.88 ± 1.89 years, and 94.8% of patients achieved anatomical success, as measured by pelvic organ prolapse quantification stage 2. The patients achieved a subjective success rate of 77.5%. The general regret rate was determined to be 6.8%. There were no reported regrets regarding sexual performance. The most prevalent postoperative complication was urinary tract infection. The study demonstrated substantial enhancements in pelvic symptom measurements between preoperative and postoperative assessments. The median Pelvic Floor Distress Inventory – questionnaire 20 decreased from 78.1 to 16.6 (*P* < .001), Pelvic Organ Prolapse Distress Inventory decreased from 45.35 to 8.3 (*P* < .001), Colorectal-Anal Distress Inventory decreased from 7.4 to 4.65 (*P* = .019), and Urinary Distress Inventory decreased from 25.2 to 6.2 (*P* < .001). All of these variables exhibited statistically significant differences.

**Conclusions:**

Colpocleisis is the most effective surgical option for older women with POP, providing high patient satisfaction, minimal regret, and significant improvements in pelvic symptoms at long-term follow-up.

## 1. Introduction

Pelvic organ prolapse (POP) can be defined as the downward herniation of pelvic organs, including the bladder, uterus, rectum, and occasionally the small intestine, as well as the anterior and posterior vaginal walls.^[1]^ POP is widespread among older women.^[2]^ Research indicates a symptomatic prevalence of 3% to 6%, while vaginal examinations show a prevalence of up to 50%. A recent extensive cross-sectional study in China reported a symptomatic POP prevalence of 9.10%, with the condition becoming more common as age increases.^[3]^ We expect a rise in the need for POP management as the global population age.^[4,5]^

POP leads to a range of bothersome pelvic floor symptoms, such as a sensation of bulging or protrusion, as well as pain and discomfort in the lower abdomen and buttocks. It is frequently associated with urinary incontinence, urinary retention, urinary tract infections, and obstructive defecation.^[6]^ As a result, women affected by POP often report a significant impact on their quality of life, as well as a negative effect on their perception of sexual attractiveness and body image.^[7]^

Surgical management of POP typically involves choosing between reconstructive and obliterative procedures. The reconstructive procedure management of POP involves a range of procedures, including hysterectomy, colporrhaphy with or without polypropylene mesh repair, and sacrospinous or sacral colpopexy, tailored to the severity of prolapse in the various vaginal compartments.^[8]^ Pelvic reconstructive surgery is particularly complex in elderly women with advanced POP, especially those at high stages (pelvic organ prolapse quantification [POP-Q] ordinal stage III).^[9]^ While the use of synthetic mesh in pelvic reconstructive surgery may help reduce postoperative recurrence rates, it must be carefully considered alongside potential drawbacks, such as longer operating times, increased blood loss, and the risk of mesh exposure, which may necessitate removal, particularly in elderly women.^[10–12]^ Many of these women have comorbid conditions, including cardiovascular diseases, chronic pulmonary disorders, impaired renal function, or long-standing diabetes mellitus, all of which contribute to a higher risk of adverse outcomes during and after surgery.^[13]^

Colpocleisis is a surgical procedure that involves vaginal obliteration, presenting a more definitive and less invasive treatment option for POP.^[14,15]^ Colpocleisis has traditionally been reserved for frail, elderly women with advanced prolapse who are considered inappropriate candidates for vaginal reconstructive procedures. Published studies have indicated that obliterative procedures, compared with reconstructive surgeries, offer shorter operative times, reduced blood loss, lower morbidity, and faster recovery.^[16]^ This procedure can be considered an appropriate option for women who are no longer sexually active or those who cannot undergo longer, more invasive surgeries.^[14,17]^

The procedure’s opponents argue that colpocleisis leads to irreversible loss of vaginal coital function, which results in considerable postoperative regret. However, studies have reported low rates of regret and a positive impact on pelvic symptoms following the procedure.^[18]^ When the uterus is kept, another controversial aspect of colpocleisis is that it is not possible to do diagnostic tests for cervical or endometrial cancer after surgery. Although the risk of endometrial cancer is generally considered too low to warrant a concomitant hysterectomy, some experts suggest evaluating the uterine cavity in asymptomatic patients prior to colpocleisis, using either ultrasound or tissue sampling.^[19,20]^

This study aims to present a retrospective analysis of colpocleisis procedures performed in our clinic. Its goal is to show the reasons for surgery, the methods used, the outcomes after surgery, and the levels of patient satisfaction and regret related to the colpocleisis surgery method used on people with POP.

## 2. Materials and methods

This study was approved by the Ondokuz Mayis University Clinical Research Ethics Committee on January 22, 2025, under application number 2025090065. The subject matter is a 1-center study examining 58 cases of colpocleisis that happened at the Ondokuz Mayis University Faculty of Medicine from July 21, 2015, to January 17, 2025. The study data were obtained from hospital automation records.

Patient data included age, body mass index, comorbidities, history of the number of vaginal deliveries, the patient’s stage of genital prolapse (the preoperative stage was classified by the POP-Q staging system), type of anesthesia, operation durations, mean estimated blood loss, postoperative discharge times, the presence of intraoperative or postoperative complications, additional procedures performed during the operation, patients monitored in the postoperative intensive care unit, follow-up period, and anatomical success. We obtained data not available from hospital records by contacting the patients.

All patients had high-stage uterine prolapse or vaginal vault prolapse in combination with advanced anterior and posterior compartment prolapse. Transvaginal sonography was performed before surgery to exclude uterine pathology. We told all patients before surgery that they could not have vaginal intercourse afterward. We obtained their informed consent.

### 2.1. Surgical technique

**Figure 1.**
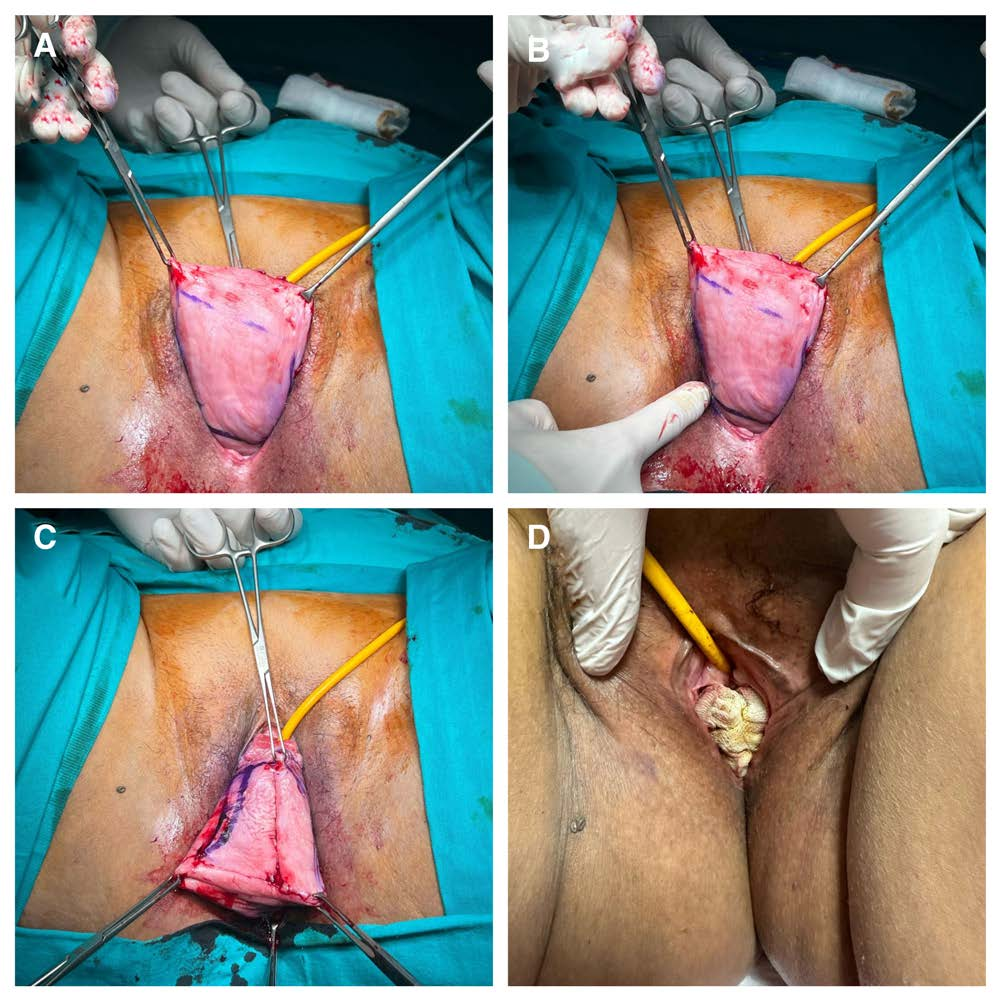
Colpocleisis operation.

Under anesthesia, we prepared the patients in the lithotomy position. Before the operation, the patients received 2 g of cefazolin sodium as a prophylactic measure. All operations were performed by the same surgeon. We sterilized the surgical field with povidone iodine solution and covered it sterilely. For thromboembolism prophylaxis, we used compression bandaging during the perioperative period. We started the operation by marking 2 rectangles in both the anterior and posterior vaginal mucosa, which we then removed after dissection. A row of imbricating sutures with delayed absorbable sutures was used to connect the muscularis layers of the front and back of the vagina. The vaginal mucosa without dissection was sutured into a tunnel for drainage purposes. After the vagina was removed, the levator ani muscle and perineal body were joined together during a perineorrhaphy. The procedure made the genital hiatus even smaller. Nine patients underwent vaginal hysterectomy using the modified Heaney technique (Fig. 1). We used the GROSS formula to calculate the mean estimated blood loss (mL).^[21]^ We determined the estimated blood volume in the GROSS formula using the NADLER formula.^[22]^

Anatomical success: We defined anatomical success in 2 different ways: There are 2 stages: POP-Q stage 1 and POP-Q stage 2.

Quality of life: The questionnaire used to measure quality of life after colpocleisis was the Pelvic Floor Distress Inventory Questionnaire-20 (PFDI-20), which is the short version of the PFDI and was developed by Barber et al in 2005. The PFDI-20 consists of 20 items and is divided into 3 subscales as follows: Pelvic Organ Prolapse Distress Inventory (POPDI-6), Colorectal-Anal Distress Inventory, and Urinary Distress Inventory (UDI-6). Total scores for each subscale range from 0 to 100, with higher scores representing a greater degree of distress or bother.

Subjective success: In our study, we used the Patient Global Impression-Improvement scale to evaluate subjective satisfaction. On the Patient Global Impression-Improvement scale, patients indicating “very much better” or “much better” are defined as “success.” We compared the preoperative and postoperative PFDI analysis results.

The study has subcategorized regret into 2 categories: “general decision regret” and “regret of coital ability.” We assessed regret by asking 1 additional question: “Do you regret choosing to have vaginal closure surgery for prolapse (Yes/No)?”

### 2.2. Statistical analysis

Data were analyzed using IBM SPSS V23 (Armonk, New York). The Shapiro–Wilk test was used to examine the conformity of the data to normal distribution. The independent samples *t* test was used to compare the parameters with normal distribution according to the presence of hysterectomy, while the Mann–Whitney U test was used to compare the parameters with normal distribution according to the presence of hysterectomy. We used Fisher exact test with Monte Carlo correction, Yates correction, and Fisher exact test to examine the relationship between categorical variables. The results were presented as mean ± standard deviation, median (minimum-maximum) for quantitative variables, and frequency (percentage) for categorical variables. We set the significance level at <0.05.

## 3. Results

Table 1 summarizes the demographic and clinical characteristics of the patients. Upon analyzing the age distribution of the patients, we found that 50% of them were under the age of 76. The ages of the patients ranged between 57 and 94 years. The mean body mass index was 26.8 ± 1.51 (calculated as weight in kilograms divided by the square of height in meters). The rate of those who had 3 or more vaginal deliveries was 91.3%. The proportion of patients with 2 or more comorbidities was 65.5%. According to POP-Q staging, the rate of patients with POP-Q apical compartment stage 4 was 72.4%, and the rate of patients with stage 3 was 20.7%.

**Table 1:** Demographic and clinical characteristics of study population.

Characteristics	Total colpocleisis (n = 58)
Age (yr)	75.55 ± 8.21 (57–94)
57–65 (%)	10.3
66–75	39.6
≥76	50
Body mass index (kg/m^2^)	26.8 ± 1.51
Number of vaginal deliveries (%)	
0–2	8.7
≥3	91.3
Medical comorbidity (%)	
Hypertension	77.6
Diabetes mellitus	22.4
2 or more comorbidities	65.5
POP-Q stage in apical compartment (%)	
0–1	1.7
2	5.2
3	20.7
4	72.4

Data are presented as mean ± standard deviation, median (range), or percentage.

The perioperative and postoperative characteristics of patients are summarized in Table 2. During the study, the mean operative time was 56.34 ± 13.73 minutes, and the estimated blood loss was 784.78 ± 436.14 mL. In our study, massive bleeding occurred in 41 (70.6%) patients as an intraoperative complication. The most frequent postoperative complication was acute urinary tract infection (12/58, 20.7%). In 18.9% of cases, additional procedures during the operation included hysterectomy. The mean postoperative hospitalization time was 3.16 ± 1.62 days. The postoperative intensive care unit monitored only 1 patient (1.7%). The mean postoperative follow-up duration was 2.88 ± 1.89 years.

**Table 2:** Perioperative and postoperative characteristics of patients.

Characteristics	Total colpocleisis (n = 58)
Operative time (min)	56.34 ± 13.73
Additional procedure (%)	
Hysterectomy	15.5
Trans obturator tape	3.4
Lymph node dissection	1.7
Intraoperative complications (%)	
Massive bleeding (blood loss > 500 mL)	70.6
Blood transfusion	0
Bladder perforation	0
Bowel injury	0
Postoperative complications (%)	
Urinary retention	0
Urinary tract infection	20.7
Thrombosis	1.7
Poor healing of vaginal incision	1.7
Postoperative hospitalization time (d)	3.16 ± 1.62
Monitored in the postoperative intensive care unit (%)	1.7
Hb level at preoperative (g/L)	12.37 ± 1.22
Hb level at postoperative 24 h (g/L)	10.7 ± 1.29
Estimated blood loss (mL)	784.78 ± 436.14
Duration of follow-up (yr)	2.88 ± 1.89

Data are presented as mean ± standard deviation, median (range), or percentage.

The postoperative success rates and regret rates of the patients are summarized in Table 3. The anatomical success rate was observed to be 82.7% (POP-Q ≤ 1). When the definition was extended to POP-Q ≤ 2, the success rate was observed to be 89.6%. In calculating subjective success using the PGI scale, patients who reported feeling “very much better” or “much better” are generally considered to be “successful.” Therefore, we calculated a success rate of 77.5% in our study. In this study, regret has been subcategorized into “general decision regret” and “regret of coital ability.” In our study, the general regret rate was found to be 6.8%, with the primary reason being POP recurrence. Our study reported no regret regarding coital ability.

**Table 3:** Evaluation of postoperative success rates and regret rates.

Parameters	Total colpocleisis (n = 58)
Anatomical success (%)	
POP-Q stage ≤ 1	82.7
POP-Q stage ≤ 2.	94.8
Subjective success (PGI-I) (%)	
Much better	24.1
Very much better	53.4
Regret rate (%)	
General decision regret	6.8
Regret of coital ability	0

Data are presented as mean ± standard deviation, median (range), or number (percentage).

PGI-I = patient global impressions—improvement.

Table 4 presents preoperative and postoperative symptom scores (1–10) during the follow-up period. The median PFDI-20 (preoperative) value was 78.1, while the median PFDI-20 (postoperative) score was 16.6, with a statistically significant difference between them (*P* < .001). The median values for POPDI-6 measurements showed a statistically significant difference (*P* < .001), with the POPDI-6 (preoperative) median value being 45.35 and the POPDI-6 (postoperative) median value being 8.3. The median value for Colorectal-Anal Distress Inventory measurements before surgery was 7.4, and after surgery, it was 4.65. There was a statistically significant difference between the median values for KRADE 8 measurements (*P* = .019). The UDI-6 (preoperative) median value was 25.2, while the UDI-6 (postoperative) median value was 6.2, with the median values for UDI-6 measurements showing a statistically significant difference (*P* < .001).

**Table 4:** Symptom scores before and after surgery (1–10 years) during the follow-up period (n = 58).

	Preoperative	Postoperative	*P*
PFDI-20	78.1 (56.7–98.8)	16.6 (4.1–100)	<.001
POPDI-6	45.35 (29.7–63.1)	8.3 (0–50)	<.001
CRAID-8	7.4 (4–17.9)	4.65 (0–46.8)	.019
UDI-6	25.2 (14.6–38.1)	6.2 (0–37.5)	<.001

CRAID-8 = Colon Rectal Anal Distress Inventory – short form 8, PFDI-20 = Pelvic Floor Distress Inventory – short form 20, POPDI-6 = Pelvic Organ Prolapse Distress Inventory – short form 6, UDI-6 = Urinary Distress Inventory – short form 6. “Wilcoxon test.’’

## 4. Discussion

Colpocleisis and other obliterative techniques represent only a small portion of the surgical options for treating POP, but evidence suggests that they are gaining increasing popularity. In the United States, obliterative procedures for POP rose from 0.77% to 2.19% of surgeries between 2002 and 2012 (*P* < .0001).^[23]^ In Canada, colpocleisis rates increased from 0.1 to 0.3 per 10,000 women between 2006 and 2016.^[24]^ The possible causes of this situation include the increasing elderly population and growing awareness about the condition. Additionally, following the Food and Drug Administration announcement regarding the use of alloplastic implants in urogynecology, their use has decreased.

The prevalence of medical comorbidities is high among older women. In our study, 65.5% of patients had 2 or more medical comorbidities, which is consistent with findings reported in previous studies.^[9,13]^

Prevention of postoperative morbidity and mortality is a major concern in the elderly surgical population. A study of 4776 patients undergoing colpocleisis in the United States found that complication rates, intensive care unit admissions, and mortality rates were low, with mean rates of 6.82%, 2.80%, and 0.15%, respectively.^[25]^ In our study, the mean postoperative discharge time was 3.16 ± 1.62 days, and 1 patient (1.7%) was followed up in the intensive care unit for 2 days postoperatively. Considering the age and comorbidity status of the patients, this rate seems tolerable. In our study, 1 patient died from a myocardial infarction 1 month after surgery. On the other hand, Zebede et al reported 4 deaths (1.3%), which included 2 cases of pulmonary embolism, 1 case of sepsis and multiorgan failure following bowel injury, and 1 case of myocardial infarction occurring 42 days post-surgery.^[13]^

The mean operation time was 56.34 ± 13.73, and the mean blood loss was 784.78 ± 436.14 in our study. In our study, 70% of the patients experienced massive bleeding as an intraoperative complication. Mcdermot et al used tranexamic acid to reduce bleeding during colpocleisis, but it was not found to be as effective as a placebo.^[25]^ However, none of them required a blood transfusion. The findings may emphasize the fragility of pelvic floor tissues, impaired coagulation function, or a bleeding diathesis in this elderly population.

Similar to other studies, the most common postoperative complication in our study was urinary tract infection (20.7%).^[26,27]^ Based on the definition used in the American College of Surgeons National Surgical Quality Improvement Program surgical risk calculator, 4.3% of the 1027 people who took part in the study got a urinary tract infection in the 30 days before surgery.^[26]^

In previous studies on this subject, the focus was more on the operation procedure and anatomical success. Recent studies have focused more on patients’ postoperative satisfaction, regret rates, and reasons. Critics of the colpocleisis procedure raise concerns that women may later regret their decision if they wish to pursue vaginal interventions and that the surgery may negatively impact their body image.^[28]^ Multiple studies have indicated that women experience minimal regret after colpocleisis.^[18,29–31]^ Additionally, patients have been shown to have sustained improvements in their postoperative body image.^[31]^

We observed a success rate of 89.6% when we extended the definition of anatomical success in our study to POP-Q 2. In early studies on this topic, the observation period was relatively short; however, more recent studies have extended it to an average of up to 5 years.^[18]^ Various studies employed different methods of subjective satisfaction assessment. In our study, the average follow-up duration was 2.88 ± 1.89 years, and according to the PGI scale used in our study, the subjective success rate was 77.5%. Our study compared PFDI-20 scores before surgery with time after surgery and found that PFDI-20 scores improved significantly after colpocleisis.

Our study also found that after follow-up, women who underwent colpocleisis had little general decision regret, at a rate of 6.8% (n = 4). Of these, 3 patients (5.1%) experienced recurrence after the first colpocleisis; 2 of these patients subsequently underwent vaginal hysterectomy and a second colpocleisis. About 10% of people who have had POP need surgery again, which is similar to or lower than the relapse rates of other native tissue repair methods.

The loss of the ability to engage in penetrative vaginal intercourse after surgery remains a significant consideration in colpocleisis. Most studies have focused on analyzing regret related to coital function. Many studies have reported no regret regarding coital ability following the procedure.^[27,30,32,33]^ One study found that a group of patients maintained sexual activity after colpocleisis through clitoral stimulation.^[34]^

The rates of concomitant vaginal hysterectomy vary across centers, but the procedure is generally associated with an increased risk of higher blood loss and prolonged operative time.^[26,35–37]^ However, reports indicate that the risk of developing endometrial cancer after LeFort colpocleisis is ~0.35%.^[38]^ In our study, we have no data indicating an increased risk of endometrial cancer after colpocleisis. Long-term follow-up of patients who did not undergo hysterectomy is ongoing. We need further studies to determine whether concomitant hysterectomy with colpocleisis is a better option than colpocleisis alone. Reports suggest that hysterectomy could potentially damage the pelvic nerves and increase the risk of urinary symptoms.^[39]^ There is no consensus on the indications and necessity of performing a hysterectomy during colpocleisis. In our study, there were 9 patients who underwent vaginal hysterectomy during colpocleisis.

A recent study comparing colpocleisis and sacrospinous ligament fixation reported shorter operative times and a longer time to recurrence surgery with colpocleisis while having similar recurrence rates to POP.^[40]^

Although the increase in mesh surgeries in recent decades has been reported as a decrease in obliterative surgeries and an increase in abdominal surgeries,^[41]^ more effective vaginal obliterative surgeries will continue to be the best option for older, sexually inactive women due to complications related to mesh and abdominal surgeries.

The limitations of this study include the low level of scientific evidence due to its retrospective design, the small sample size, and the lack of a control group. The collection of all cases from a single tertiary hospital results in a relatively small sample size from a specific region, which is another significant limitation. Another limitation is that the assessment of regret about sexual activity relies on the patient’s subjective information. The strengths of our study include a long follow-up period of an average of 2.8 years after the procedures and the use of validated questionnaires to assess each participant both pre- and postoperatively.

## 5. Conclusions

In conclusion, our study indicates that colpocleisis is the most effective surgical option for elderly women who lack sexual desire, as it has a high subjective success rate, offers high patient satisfaction in long-term follow-up, results in minimal regret, and leads to significant improvement in pelvic symptoms. Colpocleisis should be the preferred choice for older women who are not sexually active, as it helps avoid the complications associated with mesh and abdominal surgeries while providing the most effective treatment for pelvic organ prolapse; therefore, it would be appropriate to include it in POP guidelines.

## Acknowledgments

The authors would like to thank the Ondokuz Mayis University Hospital administration and the Department of Obstetrics and Gynaecology.

## Author contributions

**Conceptualization:** Çağlanur Yildiz, Ayşe Zehra Özdemir, Recep Erin.

**Data curation:** Çağlanur Yildiz, Burak Barutçu, Fatmanur Mollahüseyinoğlu Küllaç, İsmail Akkafa, Recep Erin.

**Formal analysis:** Burak Barutçu, İsmail Akkafa, Recep Erin.

**Investigation:** Çağlanur Yildiz, Burak Barutçu, Fatmanur Mollahüseyinoğlu Küllaç, İsmail Akkafa.

**Methodology:** Çağlanur Yildiz, İsmail Akkafa.

**Project administration:** Ayşe Zehra Özdemir.

**Resources:** Çağlanur Yildiz, Ayşe Zehra Özdemir, Fatmanur Mollahüseyinoğlu Küllaç, İsmail Akkafa.

**Software:** Fatmanur Mollahüseyinoğlu Küllaç.

**Supervision:** Ayşe Zehra Özdemir.

**Validation:** Çağlanur Yildiz.

**Writing – original draft:** Ayşe Zehra Özdemir, Kübra Baki Erin, Recep Erin.

**Writing – review & editing:** Kübra Baki Erin, Recep Erin.
